# Double-edge sword roles of iron in driving energy production versus instigating ferroptosis

**DOI:** 10.1038/s41419-021-04490-1

**Published:** 2022-01-10

**Authors:** Shuping Zhang, Wei Xin, Gregory J. Anderson, Ruibin Li, Ling Gao, Shuguang Chen, Jiajun Zhao, Sijin Liu

**Affiliations:** 1 Medical Science and Technology Innovation Center, Shandong First Medical University & Shandong Academy of Medical Sciences, Jinan, Shandong 250117 China; 2 Iron Metabolism Laboratory, QIMR Berghofer Medical Research Institute, Brisbane, Queensland 4006 Australia; 3 School for Radiological and Interdisciplinary Science, Soochow University, Suzhou, Jiangsu 215123 China; 4 Department of Endocrinology, Shandong Provincial Hospital, Shandong First Medical University, Jinan, Shandong 250031 China; 5 Department of General Surgery, Peking Union Medical College Hospital, Chinese Academy of Medical Sciences & Peking Union Medical College, Beijing, China; 6 State Key Laboratory of Environmental Chemistry and Ecotoxicology, Research Center for Eco-Environmental Sciences, Chinese Academy of Sciences, Beijing, 100085 China

**Keywords:** Cell biology, Biochemistry

## Abstract

Iron is vital for many physiological functions, including energy production, and dysregulated iron homeostasis underlies a number of pathologies. Ferroptosis is a recently recognized form of regulated cell death that is characterized by iron dependency and lipid peroxidation, and this process has been reported to be involved in multiple diseases. The mechanisms underlying ferroptosis are complex, and involve both well-described pathways (including the iron-induced Fenton reaction, impaired antioxidant capacity, and mitochondrial dysfunction) and novel interactions linked to cellular energy production. In this review, we examine the contribution of iron to diverse metabolic activities and their relationship to ferroptosis. There is an emphasis on the role of iron in driving energy production and its link to ferroptosis under both physiological and pathological conditions. In conclusion, excess reactive oxygen species production driven by disordered iron metabolism, which induces Fenton reaction and/or impairs mitochondrial function and energy metabolism, is a key inducer of ferroptosis.

## Facts

- Point 1. Iron plays a key role in inducing ferroptosis.
- Point 2. An imbalance in energy metabolism is closely associated with ferroptosis.
- Point 3. Iron is involved in the metabolism of glucose, lipids, and amino acids, and is thus associated with energy metabolism.
- Point 4. Disordered iron homeostasis, deregulated energy production, and ferroptosis are implicated in a variety of diseases and pathological conditions.

## Open Questions

- How much iron is required to trigger ferroptosis? Where is the threshold? Does iron overload increase the sensitivity of ferroptosis?
- Does copper possess the potential to function as an iron substitute in inducing ferroptosis?
- How does lipid peroxidation lead to cell death?
- How do dysregulated glucose, lipid, and protein metabolism processes contribute to ferroptosis?
- What role does mitochondria play in ferroptosis induction?
- One form of regulated cell death would dominate other forms at a specific disease stage. How is this mediated?

## Introduction

Iron is necessary for almost all forms of life, ranging from bacteria to humans. It contributes to a number of essential biological processes, including DNA replication, the tricarboxylic acid cycle (TCA), and electron transport in mitochondria [1]. However, iron can also catalyze reactions that lead to the production of toxic reactive oxygen species (ROS) (e.g., the Fenton reaction) [2, 3]. Therefore, iron homeostasis is tightly controlled at both the cellular and systemic levels by a complex network of regulatory signaling pathways, and disturbances in iron homeostasis are linked to a range of pathologies. Iron deficiency can limit iron availability for heme synthesis and other biochemical pathways in multiple tissues, ultimately leading to anemia, reduced work capacity, and developmental retardation. Excess iron is also detrimental to the body, and is associated with an increased risk of cancer, neurodegenerative diseases, and diabetes [4].

Although the aspects of iron-mediated cell death were established many years ago, it has only been during the last 10–20 years that ferroptosis, a specific, iron-dependent form of regulated cell death (RCD), has been recognized as a discrete entity [5]. Unlike apoptosis, necroptosis, and pyroptosis, ferroptosis is characterized by two major features, lipid peroxidation and iron dependency [3, 6]. When ferroptosis is induced by exogenous or internal stimuli, polyunsaturated fatty acids (PUFAs) are oxidized by intracellular ROS that arise from iron-dependent Fenton reactions. The resulting lipid peroxides trigger cell death [6]. Although numerous studies have examined the mechanisms of ferroptosis over the last decade, many aspects of this process remain unresolved. Important questions include: How much iron is required to trigger ferroptosis? How does lipid peroxidation lead to cell death? What role do intracellular organelles, particularly mitochondria, play in ferroptosis induction? Consequently, it is of interest to elucidate the role of iron in driving energy production and instigating ferroptosis. It is of great significance to investigate the involvement of disordered iron homeostasis in the imbalanced metabolism of glucose, lipids, and amino acids, as this leads to impaired energy production and ferroptosis. Importantly, the role of ferroptosis in normal physiological processes needs to be established, and its contribution to various pathological conditions (e.g., metabolic disorders) warrants to be investigated.

The current review describes the interrelationships between ferroptosis and energy metabolism with an emphasis on the intermediate role of iron, and the role of these processes under normal and diseased conditions.

## Iron homeostasis and iron dependence in modulating energy homeostasis

### Iron and its regulation

**Fig. 1:**
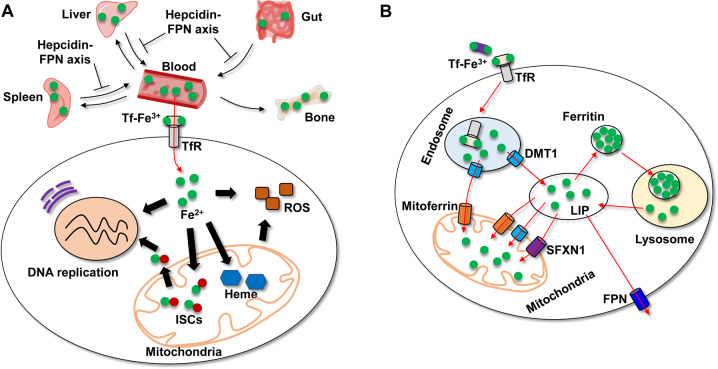
Systemic iron metabolism and iron-mediated physiological functions. **A** Systemic iron metabolism is regulated by the hepcidin-FPN axis. After the uptake of dietary iron in the gut, iron binds to circulating Tf and is transferred to organs throughout the body, particularly to the bone marrow for hemoglobin synthesis and RBC production. Liver-secreted hepcidin serves as a master regulator of systemic iron homeostasis through inducing degradation of FPN, a protein that is ubiquitously expressed and is currently the only known mammalian iron exporter. Kupffer cells in the liver and macrophages in the spleen phagocytize the damaged or aged red blood cells and recycle their iron. Iron is taken up by cells via the Tf/TfR1 pathway and used for multiple functions including heme and ISC synthesis in mitochondria, and DNA replication. ISCs are important for mitochondrial functions and DNA replication. However, excess iron can also generate ROS. **B** Iron uptake, redistribution, and export at the cellular level. Endocytosis of Tf-TfR1 complex releases iron to the cytosol LIP by DMT1 or directly to mitochondria via DMT1 and mitoferrin. Iron from LIP can be transported to mitochondria by DMT1, mitoferrin, and SFXN1, exported by FPN, or stored in ferritin. After degradation of ferritin in the lysosomes, iron is released to replenish LIP.

In the form of ferrous iron (Fe^2+^), heme, or iron sulfur clusters (ISCs), iron acts in the catalytic centers of a number of important enzymes, such as ribonucleotide reductase and DNA helicase during DNA replication, nitric oxide synthases in governing second messenger transduction, cytochrome oxidases in electron transport, and the TCA for oxidative phosphorylation (OXPHOS) and energy production. ISCs are integral components of the electron transport chain (ETC), and function as electron-transfer groups in the one-electron redox processes required for adenosine triphosphate (ATP) synthesis in mitochondria [1]. ISC-dependent electron transfer is also a prominent source of endogenous ROS production within the mitochondria (Fig. 1A). Under normal conditions, ROS are crucial for orchestrating cell physiology and signal transduction; however, overproduction of ROS results in oxidative stress and its consequent adverse effects on cells [2, 3].

Given the importance of iron in the context of fundamental physiology, the levels of iron both within cells and in the body as a whole are stringently regulated, and the processes of iron absorption, transfer, storage, and retrieval by multiple mechanisms are finely balanced (Fig. 1A). In mammals, iron is absorbed through the enterocytes of the duodenal mucosa in the gastrointestinal tract by divalent metal transporter 1 (DMT1), which provides the primary pathway for the entry of dietary iron into enterocytes [7, 8]. After being transferred from the enterocytes into bloodstream via ferroportin (FPN) [9], iron binds to transferrin (Tf) which delivers it to cells throughout the body. These cells take up iron-laden Tf via transferrin receptor 1 (TfR1). After internalization of the Tf-TfR1 complex into endosome, iron is released from Tf, and transferred to the cytosol by DMT1 to join the labile iron pool (LIP). Endosomal iron can be directly delivered to the mitochondria via the interaction between DMT1 and mitoferrin. Excess iron from LIP is stored in ferritin, which can be delivered to and degraded in lysosomes, and this, in turn, replenishes LIP. Furthermore, iron from LIP can be transferred in mitochondria by DMT1, mitoferrin, and siderofexin (SFXN1). Iron efflux is mediated by FPN (Fig. 1B) [10].

Certain cells such as immature erythroid cells in the bone exhibit particularly high iron requirements, while other cells, particularly hepatocytes and splenic macrophages, play a major role in iron storage [11]. Systemic iron metabolism is finely regulated through multiple mechanisms, including transcriptional, translational, post-translational (e.g., ubiquitin-proteasome-mediated protein degradation), and hormonal mechanisms [12]. Hepcidin-mediated FPN internalization and degradation (the hepcidin-FPN axis) is the most important regulatory mechanism for systemic iron metabolism and regulates both dietary iron intake and iron recycling from senescent red blood cells by macrophages (Fig. 1A) [12]. Quantitatively, the latter is significantly more important [12].

To this end, iron is vital for a wide range of physiological functions and processes, including DNA replication, the TCA cycle, ETC-driven ATP production, and signal transduction. In view of this, systemic and cellular iron homeostasis is finely tuned to avoid iron overload or iron deficiency through multiple regulatory mechanisms.

### Involvement of iron in the metabolism of glucose, lipid, and amino acids

Both iron deficiency and iron overload have been associated with dysregulated glucose metabolism (Table 1). Animals with iron deficiency exhibit hyperinsulinemia, hyperglycemia, and hyperlipidemia, ultimately leading to their preferential fuel usage being changed from fat to glucose [13]. Additionally, cardiomyocytes and muscle cells that are treated with iron chelators markedly increase their glucose uptake and transport, and this is associated with an increased expression of GLUT1 [14]. Conversely, iron overload decreases insulin sensitivity and induces insulin resistance, which is associated with reduced glucose uptake, and this occurs either by promoting ROS production or impairing autophagy [15]. However, an in vivo study demonstrated that mice fed a high-iron diet exhibited enhanced glucose uptake and elevated AMP-activated protein kinase (AMPK) activity in skeletal muscle and the liver [16]. These reports suggest that glucose is the preferred metabolic fuel when iron homeostasis is disturbed. Hepatic production of glucose-6-phosphatase (G6Pase), an enzyme that catalyzes the last step in gluconeogenesis, is known to be inhibited by both insulin and AMPK [17]. AMPK can be activated by iron overload, thus supporting the view that iron acts as a suppressor of gluconeogenesis. Furthermore, the transcription of gluconeogenic genes, including G6Pase, can be downregulated by heme or heme-derived iron [18].

**Table 1:** Involvement of iron in the metabolism of glucose, lipid, and amino acids.

Metabolism	Altered processes	Refs
Glucose	Iron deficiency induces preferential usage of glucose, and increases glucose uptake and transport. Iron overload induces insulin resistance and suppresses gluconeogenesis.	[13, 14, 17, 18, 147]
Lipid	Iron deficiency impairs fatty acid oxidation and desaturation of fatty acids, but promotes lipogenesis. Iron overload also inhibits fatty acid oxidation.	[20–22, 24, 26, 148]
Amino acids	Iron is required for 4-hydroxyproline synthesis, cysteine catabolism, and glycine cleavage. Ironically, both iron-driven Fenton reaction and iron chelation promote amino acid oxidation.	[27–31]

Carnitine palmitoyl transferase 1 (CPT-1) is the rate-limiting enzyme in fatty acid oxidation and conjugates fatty acids with carnitine [19]. In the fetal liver, iron deficiency markedly decreases the abundance of CPT-1 mRNA, thus suggesting that fatty acid oxidation is impaired [20]. Peroxisome proliferator-activated receptors are key transcription factors that regulate the expression of enzymes involved in fatty acid oxidation. Hepatic expression of peroxisome proliferator-activated receptor α is dramatically inhibited by iron overload. More importantly, hydroxyl radicals (produced through iron-driven Fenton/Haber−Weiss reactions) and nitrate anions (catalyzed by peroxynitrate) participate in the oxidation of multiple unsaturated fatty acids [21]. Iron deficiency and iron chelation promote fatty acid synthesis and cytosolic lipid droplet accumulation, which is accompanied by a rapid increase in intracellular citrate concentrations [22], leading to non-autophagic and non-apoptotic cell death in human breast cancer cells [23]. Both in vitro and in vivo studies have demonstrated that hepatic lipogenesis is enhanced by iron deficiency [24]. Iron is a critical component of cytochrome, ∆-6 desaturase and stearyl CoA desaturase, and desaturase activity in the liver of rats fed with low-iron diets was significantly decreased [25]. As a result, hepatic phospholipids in iron-depleted rats possessed lower proportions of palmitoleic and oleic acids and a higher proportion of stearic acid [26], thus indicating impaired desaturation of saturated and essential fatty acids.

An important amino acid, 4-hydroxyproline, in collagen is synthesized from proline by the iron-containing dioxygenase prolyl-4-hydroxylase [27]. Cysteine dioxygenase, another iron-containing enzyme, is vital for cysteine catabolism [28]. BOLA3, a ISC biogenesis protein, is required for glycine cleavage, and BOLA3 deficiency leads to increased glycine accumulation and promotes endothelial proliferation [29]. Furthermore, the iron-driven Fenton reaction catalyzes the oxidative deamination-decarboxylation of all amino acids, with [Fe(III)(salen)]Cl serving as an active and selective catalyst for the oxidation of amino acids [30]. Importantly, NH_4_, α-ketoacids, CO_2_, aldehydes, and carboxylic acids are generated by the oxidation of amino acids via the Fenton reaction [31]. The oxidation of amino acids is also promoted by iron chelators [31]. Although our knowledge of the involvement of iron in amino acid metabolism is relatively limited, it is clearly an area that warrants further investigation (Table 1).

### The convergence of metabolic activities and their association with iron

**Fig. 2:**
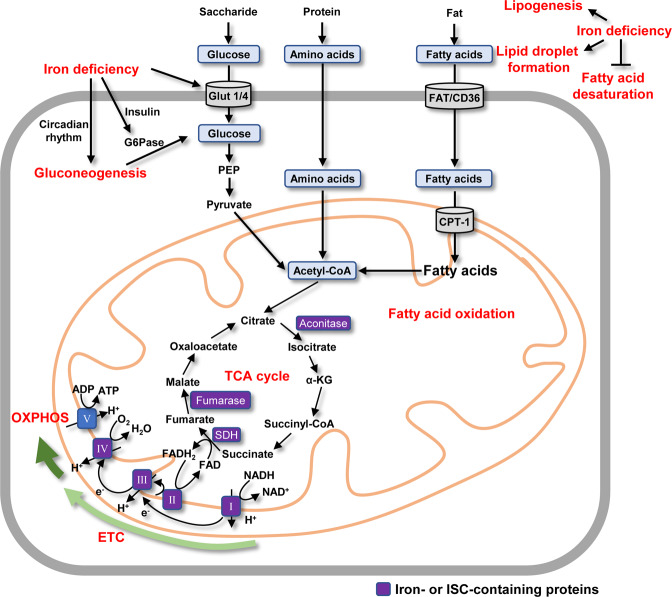
Roles of iron in the metabolism of glucose, lipids, and amino acids and, in energy production. Iron deficiency can increase intracellular glucose levels by promoting glucose uptake and gluconeogenesis. Lipid metabolism is also altered in response to iron deficiency through increased lipogenesis and lipid droplet formation, and the inhibition of fatty acid desaturation. The metabolism of glucose, lipids, and amino acids converges in mitochondria at the point of acetyl-CoA, which enters the TCA cycle for energy production. Iron levels can also modulate the synthesis of several key enzymes in the TCA cycle, including aconitase, SDH, and fumarase, and complexes I, II, III, and IV in the ETC.

Energy production from glucose catabolism is conducted by two major metabolic programs, anaerobic glycolysis in the cytoplasm and aerobic OXPHOS in mitochondria. Glycolysis links the metabolism of glucose, lipids, and amino acids. Glucose is converted into pyruvate by enzyme-catalyzed reactions in the cytoplasm, and it then enters the mitochondria and is decarboxylated to form acetyl CoA. Under aerobic conditions, acetyl-CoA enters the TCA cycle and is oxidized to water and carbon dioxide, ultimately producing a large amount of ATP through OXPHOS (Fig. 2).

Iron is essential for metabolic activity in all living organisms due to its catalytic role. In the TCA cycle, ISCs are crucial cofactors for three enzymes, aconitase, succinate dehydrogenase (SDH), and fumarase (Fig. 2) [32]. Intriguingly, there is also a cytosolic form of aconitase, and when it loses its ISC, it becomes iron regulatory protein 1 that acts as an important regulator of cellular iron uptake and storage [33]. Given the importance of iron for these various enzymes, modulating iron levels has the potential to alter the expression of enzymes involved in glycolysis and the TCA cycle, including citrate synthase, aconitase, isocitrate dehydrogenase, and SDH and also their intermediates [34]. ISCs are also essential for OXPHOS efficiency as a key component in several complexes in the respiratory chain, including complex I (NADH-dehydrogenase), complex II (SDH), and complex III (ubiquinol: cytochrome *c*-oxidoreductase) [32]. Impaired ISC biogenesis and assembly lead to deficiencies in multiple respiratory chain complexes [35].

Excess iron alters the mitochondrial oxidative enzymatic machinery, and iron-guided metabolic remodeling is gaining increasing attention. For example, iron supplementation results in pyruvate accumulation and a decrease in lactate levels in conjunction with changes in the concentrations of several other metabolites [34]. Iron overload reduces glucose oxidation in murine cardiac muscle, and this is accompanied by decreased activity of mitochondrial complexes I-IV and low ATP production [36]. In contrast, iron deprivation enhances glycolysis and abolishes OXPHOS in human macrophages, along with associated inhibition of the TCA cycle [22]. Metabolic reprogramming has also been observed in human fibroblasts and cardiac myocytes when iron status is perturbed [37].

## Ferroptosis and its dependence on iron

### Discovery and regulatory network of ferroptosis

In 2003, a novel synthetic compound, erastin was found to initiate a new form of non-apoptotic cell death in RAS-overexpressing cancer cells [38]. Subsequently, erastin was observed to directly bind to mitochondrial voltage-dependent anion channels, leading to oxidative stress and cell death via a non-apoptotic mechanism in cancer cells with oncogenic RAS [39]. Furthermore, Ras-selective lethal small molecule 3 (RSL3) was revealed to induce this type of cell death with the implication of labile iron [40]. In 2012, this new form of RCD was formally named ferroptosis by Dixon et al. [5]. Ferroptosis is characterized by iron-dependent ROS accumulation, glutathione (GSH) depletion, glutathione peroxidase 4 (GPX4) suppression, and ultimately lipid peroxidation [3, 6]. The morphological features of ferroptosis include intact nuclei and aberrant mitochondria with a decrease in the number of mitochondrial cristae, and the occurrence of inner membrane condensation, outer membrane rupture, and mitochondrial shrinkage [41].

**Fig. 3:**
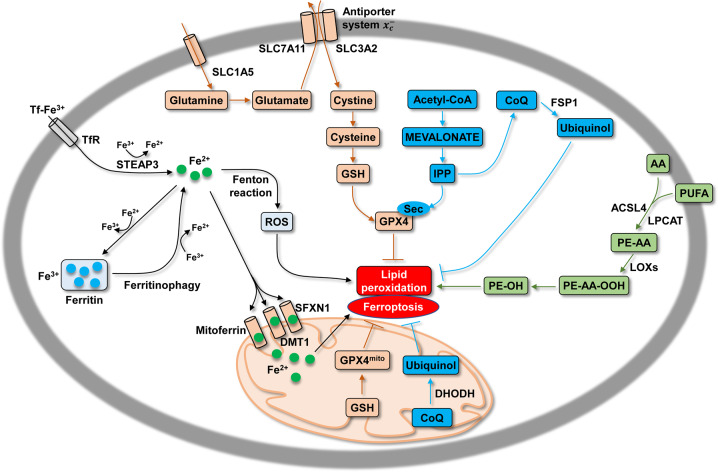
Overview of the metabolic routes contributing to ferroptosis. Several metabolic pathways are involved in the regulation of ferroptosis, including: (i) iron-Fenton reaction (black). ROS are produced by the Fenton reaction that is driven by excessive iron, which can be derived from the import of extracellular iron and the supply of intracellular stored iron via ferritinophagy. (ii) GPX4 antioxidant activity (brown and blue). Suppression of lipid peroxidation and ferroptosis occurrence largely depends on GPX4 activity, which relies on GSH and IPP-derived Sec. Cystine import by the amino acid antiporter system $x_c^ -$ and mevalonate routes are necessary for GSH and IPP production, respectively. Additionally, IPP-derived CoQ inhibits ferroptosis mediated by FSP1 in the cytosol or by DHODH in mitochondria. (iii) Lipid metabolism pathway (green). The oxidation of PUFA and AA is also involved in ferroptosis.

Ferroptosis can be induced or inhibited through several metabolic pathways (Fig. 3). After Tf-TfR1-mediated import and STEAP3-mediated conversion of Fe^3+^ to Fe^2+^, free Fe^2+^ can catalyze the production of ROS through the Fenton reaction, followed by lipid peroxidation and the induction of ferroptosis [2]. Degradation of ferritin (the major cellular iron storage protein), a process termed ferritinophagy, also provides free Fe^2+^, which contributes to ferroptosis [42]. Furthermore, increased cytoplasmic Fe^2+^ via ferritinophagy was observed to activate SFXN1 expression on mitochondrial membrane. SFXN1, in turn, transported Fe^2+^ from cytoplasm into the mitochondria, leading to mitochondrial ROS induction and ferroptosis in sepsis-induced cardiac injury [43]. Apelin-13 can activate the expression of SFXN1 and nuclear receptor coactivator 4 (NCOA4), thus leading to ferroptosis via ferritinophagy and transport of cytoplasmic Fe^2+^ into mitochondria [44]. DMT1 is also expressed in the outer mitochondrial membrane and induces mitochondrial uptake of iron and manganese [45], thus indicating its potential role in iron influx and ferroptosis.

The antioxidant activity of GPX4 is key to inhibiting lipid peroxidation and preventing ferroptosis by restoring cellular redox homeostasis [46]. Cystine-derived GSH is necessary for the maintenance of GPX activity. While GSH can be produced from cysteine and glutamate, extracellular cystine is imported by the amino acid antiporter system $x_c^ -$, that consists two components, solute carrier family 7 member 11 (SLC7A11) and solute carrier family 3 member 2 (SLC3A2). System $x_c^ -$ exchanges cystine with intracellular glutamate [47]. Glutamate itself can be replenished by glutamine import via solute carrier family 1 member 5 (SLC1A5) [48]. Isopentenyl pyrophosphate (IPP)-derived Sec is also necessary for the catalytic activity of GPX4. IPP can be produced from acetyl-CoA via the mevalonate pathway and function as the donor of Sec in the incorporation of GPX4 [49]. Erastin and its derivatives induce ferroptosis by inhibiting cystine import by the system $x_c^ -$ [50]. RSL3 directly inhibits GPX4 [41]. Furthermore, IPP-derived CoQ can be converted into ubiquinol (CoQH_2_) by ferroptosis suppressor protein 1 (FSP1) to inhibit lipid peroxidation and ferroptosis [51], and FSP1 can act in parallel to the GPX4 pathway to inhibit ferroptosis in cancer cells [52]. Interestingly, conversion of CoQ to ubiquinol by dihydroorotate dehydrogenase (DHODH) in mitochondria was recently reported to suppress mitochondrial lipid peroxidation and ferroptosis [53]. More importantly, DHODH operates in parallel with mitochondrial GPX4 to inhibit ferroptosis, and this is independent of cytosolic FSP1 and GPX4 [53]. PUFA and arachidonic acid (AA) can be converted into PE-AA by lysophosphatidylcholine acyltransferase (LPCAT) and acyl-CoA synthetase long-chain family member 4 (ACSL4). PE-AA is then oxidized by lipoxygenases (LOXs), leading to ROS production and lipid peroxidation [54].

Ceruloplasmin and hephaestin are two multicopper ferroxidases that play important roles in iron export. Knockout of ceruloplasmin and hephaestin was demonstrated to induce iron deposition in mouse astrocytes and oligodendrocytes, respectively [55, 56]. Furthermore, a copper chelator, cuprizone, was revealed to induce rapid ferroptosis-mediated loss of oligodendrocytes by mobilizing iron from ferritin [57].

### The physiological roles of ferroptosis

Little is known regarding the physiological functions of ferroptosis. GPX4 deficiency, which predisposes cells to ferroptosis, was determined to be embryonically lethal in mice [58], and GPX4 deficiency resulted in impaired antiviral defenses [59], neurodegeneration [60], and enhanced ischemia/reperfusion injury [61]. GPX4 ablation in the hematopoietic system resulted in anemia as a result of failed maturation of reticulocytes into red blood cells [62]. These reticulocytes accumulated large autophagosomes that engulfed the mitochondria [62], thus suggesting an indispensable role for GPX4 in erythropoiesis. Ferroptosis has also been demonstrated to be activated to combat the infection of mice with *Plasmodium falciparum* [63] and rice with the fungus *Magnaporthe oryzae* [64]. Additionally, ferroptosis has been revealed to play a critical role in the defense against tumorigenesis. P53 and BAP1, two important tumor suppressors, predispose nascent tumor cells to ferroptosis by downregulating SLC7A11 expression [65]. Cells carrying a p53 mutation that was defective in apoptosis induction were revealed to be capable of suppressing tumorigenesis by potentiating ferroptosis [65]. Additionally, ferroptosis was determined to be involved in tumor suppression by CD8+ cytotoxic T lymphocytes, as demonstrated by ferroptotic cell death in mouse melanomas [66].

However, little is known regarding the physiological roles of ferroptosis during ageing. In the roundworm *Caenorhabditis elegans* (*C. elegans*), glutathione depletion is inversely correlated with the aging-related accumulation of ferrous iron, thus leading to the priming of ferroptosis [67]. Inhibition of ferroptosis can reduce age-related cell death, thus increasing the lifespan of *C. elegans*. The contribution of ferroptosis at specific life phases rather uniformly throughout life appears to be particularly important in determining the lifespan of *C. elegans* [67]. Other areas where ferroptosis may be important include placenta shedding, a process in which iron accumulation occurs [68].

In summary, ferroptosis may contribute to embryonic development, erythropoiesis, determination of lifespan, and defense against infection and tumors under physiological conditions. How iron is involved and contributes to ferroptosis under these diverse circumstances remains poorly understood. Thus, both the activation and inactivation of ferroptosis are indispensable in the context of physiological settings.

### A key role of iron in ferroptosis

**Fig. 4:**
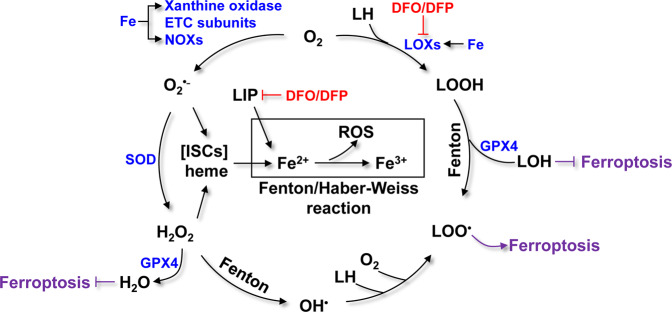
Iron-driven ROS generation and lipid oxidation in ferroptosis. O_2_^•−^ is generated from iron-containing proteins, including xanthine oxidase, ETC subunits, and NOXs. O_2_^•−^ can be further converted to H_2_O_2_. Both O_2_^•−^ and H_2_O_2_ attack ISCs and heme, leading to the release of Fe^2+^. The Fe^2+^-driven Fenton reaction generates OH^•^, which can react with LH to generate LOO^•^. Meanwhile, LH can be oxidized by LOXs to produce LOOH, which can be further converted into LOO^•^ by the Fenton reaction. Finally, LOO^•^ induces ferroptosis. Ferroptosis can be inhibited by iron chelators (e.g., DFO and DFP) and GPX4.

ROS generation through the iron-catalyzed Fenton/Haber−Weiss reaction is an essential step in ferroptosis [2, 3] (Fig. 4). Superoxide radicals (O_2_^•−^) can be generated from O_2_ by iron-containing proteins, including cytochrome P450 enzymes, NADPH oxidases (NOXs), and subunits of the mitochondrial ETC [2]. O_2_^•−^ can be further reduced to H_2_O_2_ by superoxide dismutase (SOD). As a consequence, O_2_^•−^ and H_2_O_2_ attack heme and proteins containing ISCs, leading to the release of reactive Fe^2+^ [69]. However, the major source of Fe^2+^ is LIP. The LIP is a pool of non-protein-bound, chelatable and redox-active iron, that serves as a source of free iron and sits at the crossroads of iron metabolism. LIP levels are primarily regulated by iron uptake, iron release from ferritin, and iron utilization. Driven by the conversion of Fe^2+^ to Fe^3+^, the Fenton/Haber−Weiss reaction generates hydroxyl radicals (HO^•^) from H_2_O_2_. HO^•^ further reacts with polyunsaturated lipids (LH), including PUFA, to generate lipid radicals (L^•^) and initiate lipid peroxidation. Following the initiation stage, a propagation stage occurs, in which L^•^ reacts with more LH to generate lipid peroxide (LOOH) and L^•^. LOOH can react with Fe^2+^ and Fe^3+^ to generate LO^•^ and LOO^•^, respectively. Iron-containing LOXs, such as arachidonate-15-lipoxygenase, also catalyze the reaction between O_2_ and LH to form LOOH [70, 71]. Moreover, iron is a component of the catalytic subunit of LOX [72]. In general, iron-dependent LOXs initiate ferroptosis, whereas the iron-driven Fenton reaction propagates ferroptosis [73].

Although these toxic ROS can be eliminated by the x^−^_c_-GSH-GPX4 and FSP1-CoQ axes (Fig. 3), excessive and continuous production of ROS may eventually induce ferroptosis. Inhibition of iron uptake by knocking down TfR1 can suppress lipid ROS formation [74]. Furthermore, compounds that chelate intracellular iron, such as desferrioxamine (DFO) or deferiprone (DFP), can suppress lipid ROS generation [5]. Iron chelators can also remove iron from LOXs, thus rescuing ferroptosis [75]. Ferritinophagy has been reported to induce ferroptosis by releasing iron from ferritin and thus increasing intracellular LIP levels (Fig. 3) [76, 77]. Knocking down NCOA4 inhibits ferritinophagy and subsequently suppresses lipid ROS formation [42]. Suppression of the mitochondrial protein frataxin, an iron chaperone that drives ISC biogenesis, has been demonstrated to promote cysteine deprivation-induced ferroptosis in cancer cells [78]. Deletion of mitochondrial iron-sulfur of the protein NEET (2Fe−2S) contributes to ferroptosis by inducing iron accumulation in mitochondria and mitochondrial lipid peroxidation [79]. Iron accumulation induced by the downregulation of mitochondrial ferritin can also cause mitochondrial ROS accumulation, leading to ferroptosis [80].

Taken together, both cytosolic iron and mitochondrial iron is an essential for ferroptosis. Iron-containing LOXs are required for initiating lipid peroxidation, whereas the iron-driven Fenton reactions are required for propagating lipid peroxidation. Ferroptosis is enhanced by the conversion of non-reactive Fe^3+^, which is primarily stored in ferritin, heme, and ISCs, into labile Fe^2+^. The inhibition of iron uptake and chelation of intracellular iron are effective in reducing lipid peroxidation and suppressing ferroptosis. However, the details of how intracellular iron levels, particularly the size of the LIP, are controlled and what threshold of iron concentration is required to induce ferroptosis remain elusive.

### Contribution of mitochondrial dysfunctions to ferroptosis

As mitochondria play an important role in ROS production, they are closely associated with ferroptosis [81]. Indeed, complete depletion of mitochondria increases the tolerance of cells to ferroptosis under cysteine deprivation conditions [81, 82]. However, cells that are only partially depleted of mitochondria remain sensitive to ferroptosis [83], thus suggesting that residual mitochondria are capable of initiating ferroptosis. The inhibition of the TCA cycle and ETC also suppresses ferroptosis, and this is consistent with the role played by mitochondria in generating ROS [81, 82]. In response to cysteine deprivation or erastin treatment, several enzymes in the TCA cycle, including fumarate hydratase (FH), aconitase (ACO), and citrate synthase (CS), are required to induce ferroptosis [81]. Renal cancer cells with FH loss are resistant to cystine deprivation-induced ferroptosis [81]. Additionally, reducing the activity of the TCA cycle suppresses lipid peroxidation and ferroptosis [84]. Consistent with the importance of this process in driving ferroptosis, inhibiting the activities of complexes I−IV of the ETC suppresses the accumulation of ROS and the induction of ferroptosis in response to either cysteine deprivation or erastin treatment [81]. Under cysteine deprivation, mitochondrial respiration is promoted, leading to ROS production, lipid peroxidation, and ferroptosis. However, regardless of cysteine depletion or erastin treatment, glutamine (Gln) is required to induce ferroptosis [81]. Moreover, fatty acid metabolism in mitochondria is an important contributor to ferroptosis by inducing lipid peroxidation [85].

### Iron-related mechanisms underlying other forms of RCD

There are several characteristics that distinguish ferroptosis from apoptosis, necrosis, and pyroptosis [5, 41] (Table 2). In particular, ferroptosis induces a series of mitochondrial morphological changes, including a reduction in the number of cristae, outer membrane rupture, and membrane coagulation, whereas other forms of RCD only exhibit swollen mitochondria [5]. However, ferroptosis shares some regulators and signaling pathways with other forms of RCDs. For example, all of these pathways can be triggered by iron-related signals (Table 2). For intrinsic apoptosis, iron-dependent oxidative stress is an important inducer of mitochondrial outer membrane permeabilization. This is followed by the release of cytochrome c and activation of caspase-9 and caspase-3, leading to the induction of apoptosis [86]. Iron-induced ROS also dissociate thioredoxin from apoptosis signal-regulating kinase 1 (ASK1). Consequently, ASK1 is activated to stimulate the c-Jun N-terminal kinase (JNK)/p38 pathway, thus leading to apoptosis [87]. In extrinsic apoptosis, excess iron prevents the generation of a short form of Fas by inhibiting the alternative splicing of Fas, and thus activates caspase-8-dependent apoptosis [88]. Similarly, suppression of alternative splicing of Fas by iron activates the mixed-lineage kinase domain-like (MLKL) and receptor-interacting serine-threonine kinase (RIPK), thus leading to necroptosis [89]. Heme-induced tumor necrosis factor α also activates the MLKL and RIPK pathways [90]. Moreover, iron-induced ROS are involved in necroptosis [91]. Iron-induced formation of ROS causes oxidation and oligomerization of Tom20, leading to the recruitment of Bax and subsequent cytochrome c release and caspase-3 activation. Caspase-3 further triggers gasdermin E (GSDME) cleavage, leading to a switch from apoptosis to pyroptosis [92].

**Table 2:** Morphological features and iron-related mechanisms for different forms of RCD.

	Morphological features	Iron-related mechanisms	Inhibitors	Refs
Apoptosis	Cell shrinkage, membrane blebbing, cytoskeletal disintegration, and formation of apoptotic bodies; chromatin condensation, DNA fragmentation, and global decay of mRNAs. No obvious alteration of mitochondria	Increased mitochondrial outer membrane permeabilization by iron-induced ROS activates caspase-9/3; iron-induced ROS dissociates ASK1 from Trx, thus leading to activation of JNK and p38; suppression of alternative splicing of Fas by iron facilitates caspase-8 activation.	zVAD-FMK	[5, 86–88]
Ferroptosis	Small mitochondria and abnormal mitochondrial membrane (increased membrane density, decreased cristea, and ruptured outer mitochondrial membrane). No obvious alteration of nucleus	Formation of lipid ROS by LOXs and iron-mediated Fenton reaction; Systemic inhibition of Xc and deactivation of GPX4 lead to failed clearance of lipid ROS; Lipid ROS induces ferroptosis dependently (cell-specific) or independently via ASK1-p38 and JNK signaling.	Fer-1, DFO, Baf-A1	[3, 5, 6, 71, 72]
Necroptosis	Plasma membrane disruption, cell and organelle swelling, moderate chromatin condensation, and leakage of cellular constituents	Activation of MLKL/RIPK through (1) suppressing alternative splicing of Fas by iron, and (2) TNF-alpha secretion by heme; iron-induced ROS.	Nec-1, NSA	[5, 89–91]
Pyroptosis	Plasma membrane bubbling and cell swelling with an intact nucleus	Iron-induced ROS causes oxidation and oligomerization of Tom20, thus leading to recruitment of Bax and subsequent cytochrome c release and caspase-3/3-GSDME signaling activation.	NAC, GSH, caspase inhibitors	[5, 92, 149]

Collectively, cytochrome c release and caspase activation driven by iron-induced ROS are the common routes for the activation of intrinsic apoptosis and pyroptosis, while the suppression of short Fas by iron is a common route for the activation of extrinsic apoptosis and necroptosis. Thus, excess iron is one of the inducers for the activation of apoptosis, necroptosis, and pyroptosis, while ferroptosis is initiated by iron-dependent LOXs and propagated by the iron-driven Fenton reaction.

## Dysregulated iron homeostasis, energy production, and ferroptosis in pathological conditions

Ferroptosis has been implicated in a variety of diseases, including cancer, diabetes, neurodegenerative diseases, and ischemia/reperfusion injury in many organs. More importantly, disorders of iron hemostasis and energy production frequently occur in these pathological conditions. Here, we review the literature to elucidate the complicated connections among iron homeostasis disorders, dysregulated energy production, and ferroptosis in the context of these pathological conditions, particularly in cancer, diabetes, and neurodegenerative diseases.

### Ferroptosis resistance in cancer cells

Many types of cancer cells appear to be intrinsically sensitive to ferroptosis. Importantly, sensitivity to ferroptosis occurs during the therapy-resistant state transitions in cancer cells [93]. Furthermore, cancer cells with a higher degree of malignancy, particularly those with high metastatic capacity, are more sensitive to ferroptosis [94]. Indeed, the levels of intracellular iron, PUFAs, oxidative stress, and lipid peroxidation are key factors in determining the susceptibility of cancer cells to ferroptosis [54, 70, 95]. However, cancer cells can also develop resistance to ferroptosis. The mechanisms underlying resistance to ferroptosis are not well defined, and several routes have been implicated.

FSP1 was observed to be capable of compensating for GPX4 deletion to inhibit ferroptosis in cancer cells [51]. Furthermore, FSP1 expression was positively correlated with ferroptosis resistance across hundreds of cancer cell lines and in mouse tumor xenografts [52]. Monounsaturated fatty acids (MUFAs) were demonstrated to potently inhibit ferroptosis in human fibrosarcoma cells after activation by ACSL3 [96]. The tumor malignancy of liver cancer is modulated by the balance between HIC1, a transcription factor controlling the expression of a set of ferroptosis-upregulated factors, and HNF4A, another transcription factor controlling the expression of a set of ferroptosis-downregulated factors [97].

Cancer cells exhibit particularly high iron requirements to support their rapid growth, and consequently, they are well adapted for acquiring iron and preventing its loss [98]. For example, TfR1 is highly expressed on the surface of cancer cells to facilitate iron uptake and support enhanced survival and resistance to chemotherapy of these cells [99]. Consistent with an increased iron content of cancer cells, levels of the iron storage protein ferritin are increased in many cancers, including breast cancer, and ferritin can be used as a prognostic marker for breast cancer progression [100]. We have previously demonstrated that serum levels of the iron regulatory peptide hepcidin are increased, while levels of its target FPN are decreased in breast cancer tissue from patients, and this is consistent with increased iron levels in breast cancer cells [100].

Importantly, disordered iron metabolism, dysregulated *p53* expression, and mitochondrial dysfunction appear to be integrated in cancer metabolic reprogramming, and this may explain cancer resistance to ferroptosis. Cancer cells adapt to hypoxia through metabolic reprogramming [101]. Glycolysis is enhanced in the cytosol, while the TCA cycle and OXPHOS are inhibited in the mitochondria of cancer cells under hypoxia in a response termed the Warburg effect [102]. Reprogrammed glucose metabolism in cancer cells is coupled with increased uptake of glucose and amino acids, reduced ATP production [103], and reduced ROS generation. This, in turn, increases the carbon supply for synthesizing proteins, lipids, and nucleic acids, prevents ROS-triggered apoptosis [103], and suppresses ferroptosis by oxidative stress. Adaption to hypoxia is primarily regulated by hypoxia inducible factors (HIFs 1-3), which are composed of α and β subunits [104]. Under hypoxia, ubiquitination and degradation of the HIF α subunits, a process that is mediated by prolyl-4-hydroxylase-catalyzed hydroxylation, is inhibited [105]. Ironically, iron is required for the activity of prolyl-4-hydroxylase [105]. P53 can activate ferroptosis by suppressing SLC7A11 [106]. P53 can also inhibit the production of some anti-ferroptosis metabolites, such as squalene and ubiquinone, by modulating the mevalonate pathway [107]. However, *p53* expression is downregulated under iron overload conditions via heme−p53 interactions [108]. Whether FSP1, MUFAs, and the disrupted balance of HIC1 and HNF4A correlate with metabolic reprogramming in inducing cancer resistance to ferroptosis still needs to be investigated. Recently, energy stress was observed to inhibit ferroptosis, and human renal carcinoma cells exhibiting high basal AMPK activation were demonstrated to be resistant to ferroptosis via AMPK-mediated phosphorylation of acetyl-CoA carboxylase and biosynthesis of PUFA [109]. However, AMPK-mediated BECN1 phosphorylation was reported to promote ferroptosis by directly blocking system $x_c^ -$ activity in human colorectal carcinoma cells [110]. Whether AMPK activation upon energy stress has a cross-talk with metabolic reprogramming to induce ferroptosis resistance also warrants investigation. Furthermore, how iron accumulation and metabolic reprogramming co-exist in cancer cells remains unknown.

**Fig. 5:**
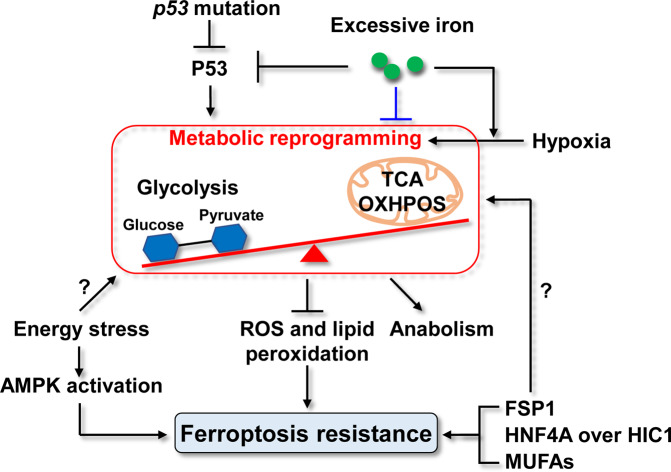
The mechanisms underlying ferroptosis resistance in cancer cells. In cancer cells, direct and indirect mechanisms may be involved in inducing ferroptosis resistance. Elevated levels of FSP1 and MUFAs, and a disrupted balance between HNF4A and HIC1 can directly inhibit ferroptosis in cancer cells. Furthermore, *p53* mutations can directly induce ferroptosis resistance. In contrast, metabolic reprogramming could indirectly and substantially induce ferroptosis resistance by integrating iron metabolism disorders, dysregulated p53 levels, and mitochondrial dysfunctions *p53* mutations and hypoxia induce metabolic reprogramming, including enhanced glycolysis in the cytosol and inhibition of the TCA cycle and OXPHOS in the mitochondria. This reduces ATP production and ROS generation, which can attenuate lipid peroxidation and lead to ferroptosis resistance. Accumulated iron in cancer cells can induce *p53* mutations and also directly promote metabolic reprogramming. Energy stress-induced AMPK activation also contributes to ferroptosis resistance in cancer cells.

To this end, ferroptosis resistance can be induced directly by elevating the levels of FSP1, HNF4A, and MUFAs, or by reducing P53 levels. Although iron accumulates in these cells, ferroptosis is suppressed and does not lead to excess ROS generation and lipid peroxidation due to metabolic reprogramming. Loss of function of *p53*, as a result of *p53* mutations and excess iron, and AMPK activation contributes to ferroptosis resistance either directly or indirectly by promoting metabolic reprogramming in cancer cells (Fig. 5). Targeting ferroptosis resistance is emerging as a promising therapeutic strategy for cancer treatment. Therapeutic strategies based on enhancing ferroptosis are being tested in some cancers, including chemical drugs and nano drugs.

### Ferroptosis occurrence in diabetes

Ferroptosis has been implicated in the pathology of diabetes. A high-fat high-sucrose diet diminished the expression and activity of GPX4 in the hypothalamus relative to a normal diet, indicating that GPX4 plays an important role in regulating metabolic signals [111]. Additionally, ferroptosis impairs islet function, and ferroptosis inhibitors can reverse this impairment. Interestingly, in isolated islets, bilirubin suppresses ferroptosis and ferroptosis-related characteristics, thus leading to reduced oxidative stress, elevated GPX4 expression, upregulated nuclear factor erythroid 2-related factor 2NRF2/heme oxygenase-1, and decreased iron levels [112]. Consequently, glucose levels normalized after bilirubin-pretreated islets were transplanted into diabetic mice [112]. Furthermore, maternal hyperandrogenism and insulin resistance activate ferroptosis in the gravid uterus and placenta [113]. Mitochondrial dysfunction is an important feature of diabetes mellitus and is a key component of ferroptosis. In most organs and tissues of patients with diabetes and also animal models of diabetes, excessive mitochondrial ROS production is observed, while other mitochondrial abnormalities, including impaired mitochondrial biogenesis and OXPHOS, disordered mitochondrial dynamics, and mitophagy, are tissue-specific [114]. In both type 1 diabetes mellitus (T1DM) and type 2 diabetes mellitus (T2DM), a switch in the energy source from glucose to fatty acids occurs. Increased uptake and utilization of fatty acids via β-oxidation further reduces glucose uptake in T2DM, thus leading to enhanced gluconeogenesis [115] and mitochondrial uncoupling [116]. The NADH/NAD ratio is increased due to complex I dysfunction and inhibition of the activities of complexes II, IV, and V in patients with diabetes and animal models [117]. Ultimately, the energy source switch and mitochondrial dysfunction lead to decreased ATP production and increased ROS generation [118]. Excessive ROS production in mitochondria can induce insulin resistance by decreasing GLUT4 levels, inducing beta-cell and mitochondrial dysfunction, promoting inflammation, and inhibiting insulin signaling pathways [119].

Diabetes mellitus is also associated with iron overload, another ferroptosis inducer. A variety of meta-analyses and systematic reviews have confirmed the relationship between iron homeostasis disorders and T2DM risk [120]. Pregnant women with gestational diabetes were also observed to exhibit significantly higher levels of serum iron, serum ferritin, and transferrin saturation, and increased fasting plasma glucose levels compared to those of pregnant women without this condition [121]. Additionally, increased serum ferritin levels were observed to be positively associated with the risk for T2DM in otherwise healthy women, and this was independent of known diabetic risk factors [122]. Consistent with these observations, iron depletion through phlebotomy can increase insulin sensitivity [123]. In streptozotocin-induced diabetic rats, an iron-restricted diet ameliorated diabetes-induced mitochondrial dysfunction and restored mitochondrial respiration and respiratory complex activity, thereby reducing oxidative stress [124]. Diabetes-driven ferroptosis reflects a switch in cellular energy sources, mitochondrial dysfunction, and iron overload, leading to reduced insulin secretion.

**Fig. 6:**
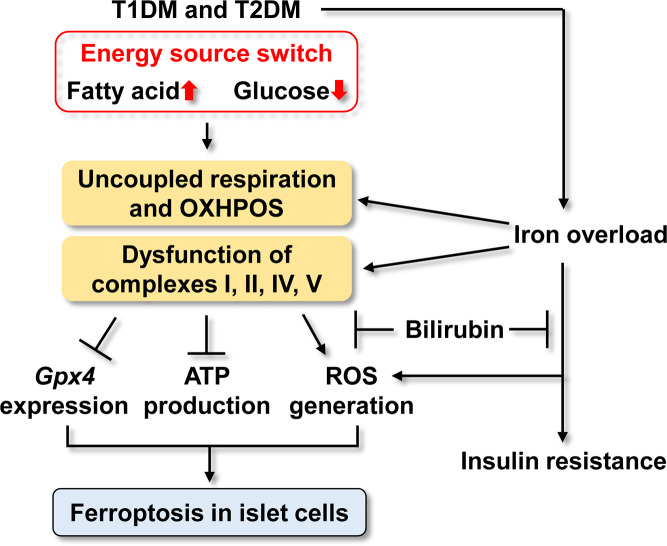
The mechanisms underlying ferroptosis induction in diabetes. In diabetes mellitus, a switch of energy source (from glucose to fatty acids) occurs, leading to uncoupling of OXPHOS, and impaired function of ETC complexes. Consequently, *Gpx* expression and ATP production are both inhibited, while ROS generation is increased. These events ultimately induce ferroptosis in the islet cells. In addition to directly promoting ROS generation and insulin resistance, iron overload in diabetes mellitus can promote uncoupling of OXPHOS and dysfunction of the components of the ETC.

Collectively, both the energy source switch and the iron overload contribute to mitochondrial dysfunction, including uncoupled respiration and reduced activities of ETC complexes. Mitochondrial dysfunction decreases ATP production, increases ROS generation, and likely suppresses GPX expression, all of which are key inducers of ferroptosis. Iron overload can directly promote ROS generation via the Fenton reaction as discussed above. Importantly, bilirubin can suppress ferroptosis in the context of diabetes by decreasing iron levels and restoring mitochondrial function (Fig. 6).

### Ferroptosis in neurodegenerative diseases

Disordered iron metabolism is closely associated with neurodegenerative diseases, and ferroptosis has been demonstrated in this context [125]. For example, upregulation of the iron regulatory protein IRP2 induces ferroptosis by increasing the intracellular iron content in aging-related auditory cortical neurodegeneration [126]. Additionally, enhanced NCOA4-mediated ferritinophagy is linked to neurodegenerative diseases, and is also linked to ferroptosis [77]. Importantly, mitochondria play an important role in regulating iron homeostasis in the brain, and are implicated in the death of neuronal cells by inducing lipid peroxidation [127].

A deficiency in complex I has been uncovered in the mitochondria of the substantia nigra (SN) and platelets from patients with Parkinson’s disease (PD) [128] and similar findings have been reported in patients with Alzheimer’s disease (AD) (deficiency of complexes I and IV) and Huntington’s disease (HD) (deficiency of complexes II and III) [129]. In patients with AD, reduced activity of various TCA cycle enzymes, including isocitrate dehydrogenase, α-ketoglutarate dehydrogenase, and pyruvate dehydrogenase, has been observed [130]. High levels of ROS are released from defective mitochondria [131], where complex I releases O_2_^•−^ to the mitochondrial matrix and complex III releases O_2_^•−^ to both sides of the IMM [132]. In turn, ROS impairs the functions of complexes I, III, and IV [133]. Furthermore, the release of cytochrome c oxidase (COX) and mitochondrial permeability are both increased by Aβ and alpha-synuclein oligomerization and polymerization in AD [134]. Nevertheless, direct evidence for the involvement of mitochondrial dysfunction in ferroptosis is still lacking in neurodegenerative diseases.

In AD, tau tangles inhibit the transport of β-amyloid precursor protein (APP), a protein that stabilizes FPN1, to the cell membrane, and in turn, this leads to intracellular iron accumulation and oxidative stress-induced cell death, including ferroptosis [135]. Iron accumulation and lipid peroxidation occur in the SN, where the death of melanized neurons is the most severe degeneration event in PD, and is associated with more rapid PD progression [136]. Furthermore, ferroptosis has been observed to be involved in the death of dopaminergic neurons, another severe degeneration event that occurs in PD [137]. DFP and ferrostatin-1 treatments were demonstrated to be beneficial to PD by chelating iron and inhibiting ferroptosis, respectively [137, 138]. Similar to AD, tau tangles in PD also inhibit the transport of APP and, in turn, they decrease the stabilization of FPN1, leading to intracellular iron accumulation and lipid peroxidation in dopaminergic neurons [139, 140]. Moreover, iron deposition in central nervous system is observed in HD and is associated disease progression [141]. Ferroptosis-related features have also been observed in patients with HD [142]. Marked lipid peroxidation has been observed in the striatal neurons of an HD mouse model [143], and a significant decrease in GSH level was observed in a HD rat model [144].

Taken together, excess iron in both the cytosol and mitochondria significantly contributes to the development of ferroptosis in neurodegenerative diseases, and this occurs primarily through its promotion of oxidative stress and lipid peroxidation. The inhibited formation of the FPN1-APP complex is the key mechanism driving iron accumulation in neurodegenerative diseases. Although neurodegenerative diseases are associated with disturbed mitochondrial function, further investigation is required to determine whether impaired mitochondria are directly involved in ferroptosis.

## Concluding remarks and perspectives

In the current review, we recapitulate the intricate functions of iron governing energy metabolism and modulating ferroptosis at the cellular and subcellular levels in the context of both physiology and pathology. Thus far, a wealth of insights has been obtained to understand the complex regulatory networks that modulate the balanced energy supply and toxic effects of excess iron on cells. In contrast, deregulated regulatory networks would give rise to iron-dependent disorders including ferroptosis and closely implicated diseases, such as cancers, diabetes, and neurodegenerative diseases. Regardless, puzzling questions and knowledge gaps still exist as follows:

i. As iron plays a key role in promoting ferroptosis, the rationale underlying the rapid involvement of iron and also the amount of iron in LIP that is sufficient for this process remained to be explored. Specifically, little is known regarding the threshold regulation of iron availability. Furthermore, whether diseases related to disordered iron homeostasis are prone to ferroptosis in certain cells remains to be tested. The difficulty in directly measuring LIP changes hinders the establishment of an iron threshold for ferroptosis. Recently, a new fluorescence resonance energy transfer iron probe (FRET Iron Probe 1, FIP-1) was designed [145] and could be used in the future to define the LIP threshold for ferroptosis. As discussed above, disordered copper metabolism induces iron deposition and ferroptosis in oligodendrocytes. Moreover, copper has also been reported to potentiate both GSH loss and nerve cell death [146], thus posing a question regarding the substitutes of iron in inducing ferroptosis.
ii. Although mitochondria have been implicated in ferroptosis, current reports remain debatable, and further efforts are warranted to elucidate the biochemical reactions related to ferroptosis in different contexts and in different cell types. Moreover, iron-mediated electron transfer, OXPHOS, and energy production converge within the mitochondria warrants to be investigated. Accordingly, it would be of great interest to untangle the unbalanced sites responsible for promoting ferroptosis.
iii. The imbalance in energy metabolism is closely associated with the occurrence of ferroptosis; however, research in this field is in its infancy, and with several questions concerning dysregulated glucose, lipid, and protein metabolism remains unresolved. For example, the current literature detailing the role of AMPK, the central sensor in response to the cellular energy state, remains controversial. This discrepancy may be ascribed to tissue specificity. Additionally, different cancers may use distinct energy sources for their major energy supply, thus indicating that their metabolic processes are different, leading to different ferroptotic signaling pathways. Additional work is required to determine the intricate nexus responsible for coordinating energy production and preventing ferroptosis.
iv. The implications of ferroptosis and iron dysregulation in metabolic disorders have not been clearly defined. For example, diabetes is often observed following iron overload, such as in patients with hereditary hemochromatosis.
v. In the real context, the co-existence of different RCD forms appears to be more frequent under physiological and particularly pathological conditions. Thus, it is important to investigate the connections among them (e.g., synergy or antagonism mode) and to clarify the common factors (such as genes, proteins, metabolites, and nutrients) and mechanisms. This would help to provide a combination therapy and solve the issues of drug resistance. However, one form of RCD would dominate other forms at a specific disease stage. How this is mediated is an interesting question. Importantly, whether a superior regulatory network coordinates these forms of RCD warrants further investigation. In this regard, iron and disordered iron homeostasis may provide a breakthrough to elaborate the interplay of ferroptosis with other iron-coupled RCD forms.

### Reporting summary

Further information on research design is available in the Nature Research Reporting Summary linked to this article.

## Supplementary information

Reporting Summary
